# Direct Imprinting of Large-Area Metallic Photonic Lattices for Infrared Polarization Filters with Broadband Tunability

**DOI:** 10.3390/nano13061022

**Published:** 2023-03-12

**Authors:** Fei Dou, Chen Peng, Miaomiao Zou, Xinping Zhang

**Affiliations:** College of Physics and Optoelectronics, Faculty of Science, Beijing University of Technology, Beijing 100124, China

**Keywords:** metallic photonic lattices, solution-processed direct imprinting, infrared plasmon resonance, fano coupling, infrared polarization filters

## Abstract

Metallic photonic lattices are promising in their application to plasmonic optical devices; however, scalable fabrication strategies are limited by sample size, response wavelength (mostly in the visible range), cost, and duration. This paper proposes a direct imprinting strategy to fabricate large-area metallic photonic lattices, which present a strong plasmonic response and broadband angle-resolved tuning properties in the infrared region. This simple fabrication strategy combines solution-synthesized Au nanoparticle colloid and imprinting technology, which does not require the use of photoresist or lithography. Thus, the feature size and response wavelength can exceed the limitations of the beam size and wave band, thereby offering the advantages of a low cost and high throughput.

## 1. Introduction

Metallic photonic lattices are promising in their application to filters [1,2], all-optical switches [3,4], energy harvesting [5,6,7], biosensor devices [8,9,10,11,12,13], and meta-materials [14,15]. However, their widespread application is restricted by their expense and low productivity of fabrication strategies, such as electron-beam lithography, focused ion-beam lithography, and direct laser writing [16,17]. These techniques allow for the fabrication of features with well-defined nanostructures; however, the operational cost of creating big areas is high, and the throughput of small substrate areas is low.

Scalable approaches, including UV lithography and interference lithography, are limited in feature size and response wavelength [17,18,19,20]. The feature size is usually below one centimeter in interference lithography fabrication because of the limited overlapped beam size, especially in the case of relatively large period fabrication, which requires a small incident angle. Furthermore, the response wavelength is usually UV because of the limited sensitivity of photoresists for both UV light and interference lithography, which means the wavelength of the incident beam is fixed. Therefore, the period of the structure (decided by the wavelength and incidence angle of the incident beam) is limited to the visible wave band. 

In recent decades, nanoimprinting lithography (NIL) has emerged as a low-cost scalable fabrication method for nanostructures, especially for polymeric materials. The first work on the NIL method was developed by Stephen Chou et al. in the 1990s, where they reported the 25-nm pattern polymer [21]. A silicon mold and a polymeric resist were used as the materials, and the experiment was performed by strongly pressing the mold (P = 500–1000 N/cm^2^) at 100 °C. This study opened the way for scalable fabrication at the nanoscale [21]. Following the emergence of soft nanoimprint lithography, first developed by Whitesides et al. in the 1990s, using polydimethylsiloxane (PDMS) elastomeric molds, the embossing pressure was sharply reduced to 50 N/cm^2^ [22]. With the improvements made in NIL afterward, this technique has been widely used in nanostructure fabrication at ambient pressures for polymers.

However, for metallic materials, the polymer NIL method cannot be directly used due to their higher melting temperatures. For example, the melting temperature of gold is 1060 °C. Therefore, high temperature (hot evaporation) and UV photoresist are still necessary to fabricate metallic nanostructure NIL technologies. 

This paper clarifies a directing imprinting strategy to fabricate large-area metallic photonic lattices which combines solution-processed Au nanoparticle colloid and NIL techniques. This strategy does not require photoresist or lithography and thus is not restricted by feature size and response wavelength limitations. Furthermore, this strategy does not require high temperatures or high beam powers. Thus, metal etching or metal oxides can be avoided. More importantly, due to the simplified procedures, this strategy can be used for a high throughput and scalable fabrication at low cost. The fabricated device presents strong plasmon resonance in the infrared region and wide angle-resolved tuning bandwidth, offering promise in application to infrared polarization filters.

## 2. Materials and Methods

### 2.1. Materials

In the fabrication, a PDMS stamp (blazing grating with the period of 1.7 μm) was used as the mold (SEM shown in Figure S1), and the Au nanoparticle colloid was used as the ink. The PDMS stamp was fabricated following the steps in [19] from a commercial blazed grating mask (GR50-0605, Thorlabs, Newtown, NJ, USA) [23]. The Au nanoparticle colloid was synthesized using the method described in [20] and resolved in p-Xylene with a 100 mg/mL concentration. The size of the synthesized Au nanoparticles is distributed in a range of 2–8 nm, following a normal distribution [20].

### 2.2. Fabrication Processes

**Figure 1:**
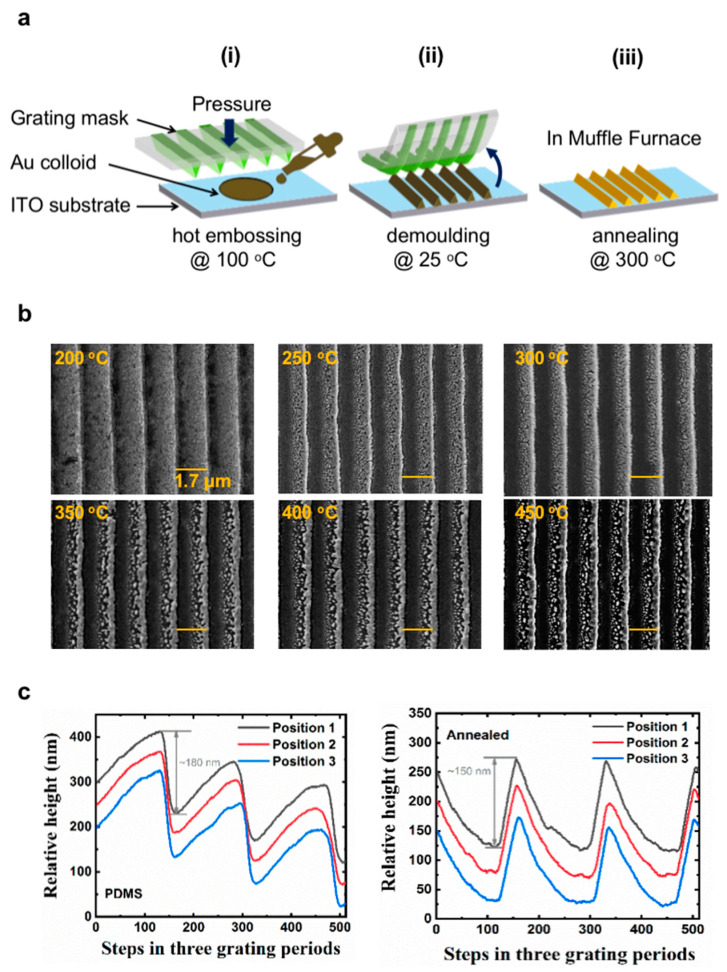
(**a**) Schematic illustration of the solution-processed direct imprinting fabrication of a blazed gold grating using a PDMS mold and Au nanoparticle colloidal solution: (**i**) hot embossing the Au nanoparticle colloid at 100 °C, (**ii**) demolding the PDMS mold at 25 °C, (**iii**) annealing the Au nanoparticle colloid film to form a gold grating in a Muffle Furnace at 300 °C. (**b**) SEM images of 1.7-μm blazed gold grating annealed at 200 °C, 250 °C, 300 °C, 350 °C, 400 °C, and 450 °C. (**c**) The surface profiles of the blazed grating on the PDMS mold and on the annealed gold wires, which are scanned in three grating periods and on three positions in a single sample.

The fabrication processes can be divided into three steps, shown in Figure 1a:

(i) Hot embossing: the Au nanoparticle colloidal (10 μL) is drop-cast on an ITO (indium tin oxide) substrate on a hot plate at 100 °C. A PDMS mold is pressed on the Au nanoparticle colloid with a pressure of 0.25 kg/cm^2^ before the colloid dries. The thickness of the grating can be tuned by altering the pressure, the volume, and the concentration of the Au nanoparticle colloidal.
(ii) Demolding: after hot embossing, the sample is removed from the hot plate to room temperature. When the temperature cools sufficiently (within about 1 min), the PDMS stamp can be demolded from the solidified Au nanoparticle colloid. The surface energy of ITO is higher than the PDMS stamp, and thus the Au nanoparticle colloid will stay on the ITO surface during demolding. The grating is therefore copied from the PDMS stamp to the solidified Au nanoparticle colloid, which will show up on top of the ITO substrate.
(iii) Annealing: the solidified Au nanoparticle colloid is annealed in a Muffle Furnace at 300 °C for 10 min to let the Au nanoparticle fully melt and aggregate. The temperature of annealing is important; when the temperature is too low (200 °C), the nanoparticles cannot be melted: note that the 200 °C image in Figure 1b is similar to that of the sample before annealing (Figure S2). Once the temperature reaches 250 °C, the Au nanoparticles melt. The SEM image shows confined structures because, after the phase transition, melted Au nanoparticles can flow within a limited space to meet other melted nanoparticles. Thus, aggregates form and solidify. Continuous Au wires can be realized via the optimization of parameters, such as embossing pressure, colloid concentration, etc. However, if the annealing temperature is too high, the agglomerate nanoparticles formed can further be melted at this temperature, which means they may flow to meet other agglomerate nanoparticles. This results in the formation of large, solidified Au-bulk, leaving space in the grooves, as illustrated in Figure 1b’s 350 °C, 400 °C, and 450 °C cases. More parameter-dependent structures are illustrated in the supporting information in Figures S3–S6.

Furthermore, the influence of the solidified Au morphology is also relative to the colloid concentration or volume per unit area, which determines the ability of melted Au to aggregate. These different annealing outcomes are the principle behind the structure distribution described in the blazed grating in Figure 1b. The volume of Au nanoparticles is different in the direction across the Au wires due to the blazed shape of the PDMS mold. At a fixed annealing temperature, the high-volume part has a high possibility to aggregate, which forms confined Au nanowires/solidified Au bulks; however, the low-volume part has a low possibility to aggregate, which forms solidified aggregates/confined Au nanowires. Thus, by adjusting the annealing temperature, we can have both confined Au nanowires and solidified Au aggregates at a single annealing temperature. Both structures can be formed on a single sample, which guarantees the formation of the blazed structure. Overall, the optimized annealing temperature of the blazed 1.7 μm gold grating in Figure 1b is around 300 °C, and there is a 21% (190 nm vs. 150 nm) loss of the height of gold wires after annealing in Figure 1c.

## 3. Results and Discussion

### 3.1. Structure Characterization

**Figure 2:**
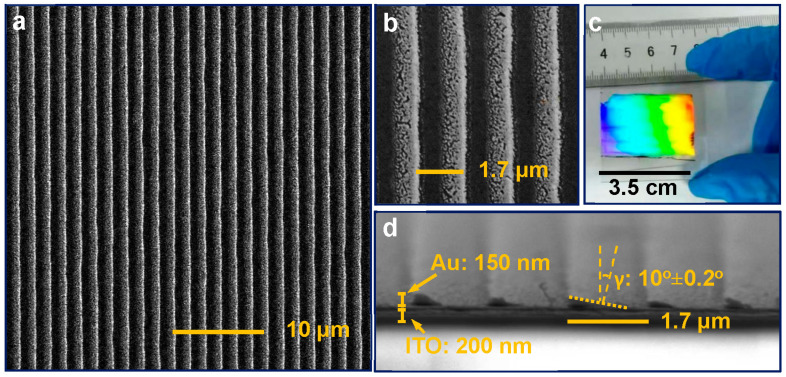
(**a**) SEM image of gold wires showing large-scale homogeneity of the blazed grating structure. (**b**) Enlarged SEM image showing the period of the blazed gold grating. (**c**) Photograph of a 1D gold grating exhibiting the scalability of our fabrication strategy. (**d**) SEM image of the cross section of gold wires displaying the structure of the blazed gold grating.

Figure 2a indicates the scanning electron microscopic (SEM) image of a finished one-dimensional (1D) periodic structure of gold wires, which has been fabricated on a 200-nm indium tin oxide (ITO) substrate. These gold wires are uniform over an area of around 40 × 40 um^2^, which is evidence of the robustness of the fabrication method. As displayed in the enlarged SEM image Figure 2b, the period of the gold wires is 1.7 um, with a duty cycle of 50%, implying the width of gold wires is about 850 nm. Meanwhile, Figure 2b shows that all Au nanoparticles have been confined completely into the grating structures, which are comprised of two parts. One part is the continuous gold wires on the right side of each wire, which each take up 1/3 of the total width of the wire; the other part is the discontinuous Au aggregates located on the left side, which take up the remaining 2/3. This differing phase distribution is a key aspect of our blazed grating fabrication strategy. It is also critical for the generation of broadband plasmon resonance. Figure 2c shows a fabricated 1D gold grating, in which the grating area is about 3.5 cm × 2.5 cm, demonstrating the scalability of our fabrication strategy. Figure 2d shows an SEM image of the cross section of the gold grating on top of the ITO substrate, demonstrating that the blazing angle γ of the grating is around 10 degrees and the height of the gold wires is around 150 nm.

### 3.2. Measurements of Polarization Selectivity and Angle Tuning Properties

#### 3.2.1. Broadband Polarization Selectivity

**Figure 3:**
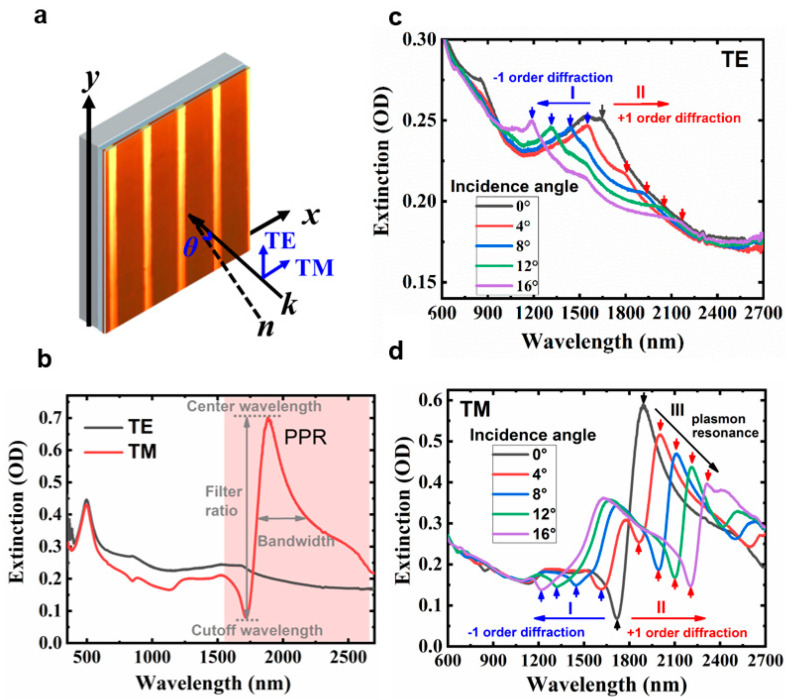
(**a**) The geometry of the incident angle and TE/TM polarization modes in the measurements. (**b**) Optical extinction spectra of the gold grating (the period is 1.7 um, annealing temperature is 300 °C, Au colloid concentration is 100 mg/mL, at a pressure of 2.5 kg/cm^2^) for the TE and TM modes with an incidence angle of 0 degrees. The center wavelength, cutoff wavelength, filter ratio, and bandwidth of the polarized filter are given in the figure. (**c**,**d**) show the optical extinction spectra for TE and TM polarization modes with the incidence angles tuned from 0 to 16 degrees. Filter parameters are shown in Table 1.

The plasmon resonance and the waveguide resonance of the fabricated gold grating are analyzed using the optical extinction spectra, which are characterized by the ratio of the transmittance of the ITO substrate, *T_substrate_*, and the transmittance of the sample, *T_sample_*, in a logarithmic scale, written as −log_10_(*T_sample_*/*T_substrate_*). A U-4100 HITACHI spectrophotometer (HITACHI Ltd., Tokyo, Japan) measured the transmittance. In the measurement, the sample is mounted on a rotation stage in the x–y plane, which can be rotated around the y-axis. Thus, the incident angle *θ* between the incident beam wave vector *k* and the surface normal of the sample *n* can be tuned, as shown in Figure 3a. For polarization, when the polarization of the incident light is parallel to the nanowires, the induced spectra are in the transverse electric (TE) polarization mode. In contrast, when the polarization of the incident light is perpendicular to the nanowires, the induced spectra are in the transverse magnetic (TM) polarization mode. The TE and TM mode excitation geometry are also shown in Figure 3a.

The broadband plasmon resonance can only be excited in the TM polarization mode due to the horizontal propagation of the plasmonic wave in the structure [20,21,22]. For this reason, we first measured the optical extinction spectra of the fabricated 1D gold grating for the TE and TM polarization modes, shown in Figure 3b, and distinguished a broad plasmon resonance band in the infrared region (1700–2700 nm) in the TM mode, which peaks at 1893 nm and has a broad bandwidth of about 258 ± 2 nm at the full width at half maxima (FWHM). The strong extinction peak contrast with the extinction valley of the TM mode in the plasmon resonance region can be applied to create an infrared polarization filter. The center wavelength of the filter can be chosen at the plasmon resonance peak at 1893 nm, and the cutoff wavelength of the filter can be chosen at the plasmon resonance valley at 1721 nm. The filter ratio is calculated by the ratio of the amplitude difference between the center wavelength and the cutoff wavelength divided by the amplitude at the center wavelength in the TM mode, as shown in Figure 3b where the result is 88.6%. This indicates our fabricated plasmonic gold diffraction grating offers a good degree of infrared polarization selection. It is worth mentioning that the extinction spectra of the opaque PDMS mold showed similar spectra on both the TM and TE modes (Figure S7).

Although the annealing temperature is important in determining the final structure of the gold grating, we found that the plasmon resonance mode can be generated even in the Au bulk and Au aggregates structures (Figures S3–S6), albeit with less intensity. This might be due to the horizontal confinement across the gold wires, which is the propagation direction of the plasmonic wave [24,25,26].

#### 3.2.2. Broadband Angle-Tuning Properties

Next, we illustrate the good angle-resolved tuning properties of our fabricated structures by investigating the angle-dependent optical extinction. It is worth mentioning that the extinction peak at 500 nm in Figure 3b is due to independent Au nanoparticles, which appear on unstructured Au nanoparticle films and are not influenced by the grating structure. Therefore, the angle-resolved extinction spectra are measured after 600 nm to emphasize the peak position shift.

Compared with the plasmon resonance mode, the waveguide resonance mode and the Rayleigh abnormal (Rayleigh scattering-induced absorption abnormal) are usually sensitive to the incident angle because of the induced optical path to the grating. To this end, we first explored the angle-resolved extinction measurement in the TE-polarized mode, shown in Figure 3c. For TE polarization, the plasmon resonance mode cannot be excited, and only the waveguide resonance mode and Rayleigh abnormal can be observed, which appear as small narrow-band extinction peaks. One can see that the extinction peak splits with increasing incident angles, displayed by downward arrows in Figure 3c: the black downward arrow at zero-degree splits to I (blue downward arrows) and II (red downward arrows), yielding two branches with increasing incidence angles. According to the diffraction function in the Rayleigh abnormal (diffraction angle is 90°) condition, *d*(*n*_air_sin*θ* ± *n*_media_sin90°) = *kλ*, where *d* is the period of the grating, *θ* is the incidence angle, and *n*_media_ could be *n*_air_, *n*_ITO_, or *n*_glass_, where *n*_air_, *n*_ITO_, and *n*_glass_ are the refractive indices of air, ITO, and glass, respectively. The extinction peak at 1721 nm (at a zero-degree incidence angle, indicated by the black downward arrow) is the Rayleigh abnormal produced by first-order grating diffraction in the air layer, which splits to the I and II branches for incidence angles larger than 0, based on the −1 and +1 order of diffraction via the grating.

For TM polarization, the plasmon resonance and the Rayleigh abnormal can both be excited. In Figure 3d, at a zero-degree incidence angle, the first-order diffraction peak in the air layer (1721 nm peak in TE mode) appears as a dip in the TM mode (downward arrow at 1721 nm). This implies strong Fano coupling between the Rayleigh abnormal and the plasmon resonance mode [27,28]. Similar to the TE polarization mode, as we increase the incidence angle, this Fano coupling signal splits into I and II branches in the TM mode because of the shifted Rayleigh abnormality based on the −1 and +1 diffraction conditions. Meanwhile, as the incidence angle increases from zero to sixteen degrees, the plasmon resonance peak shifts from 1893 nm to 2314 nm with a FWHM larger than 200 nm (branch III), as listed in Table 1. This broad spectroscopic response enables our fabricated metallic photonic lattices to act as good angle-resolved tuning infrared polarization filters. The parameters of a filter with different incidence angles are presented in Table 1. Overall, the plasmonic response in our fabricated structure is highly sensitive to the wavelength and incidence angle, which implies we can collect the TM polarization in a selected band efficiently and conveniently. For comparison, we also list the reported parameters of some other infrared polarization filters in Table 2.

### 3.3. Metallic Photonic Lattices with Different Structures

**Figure 4:**
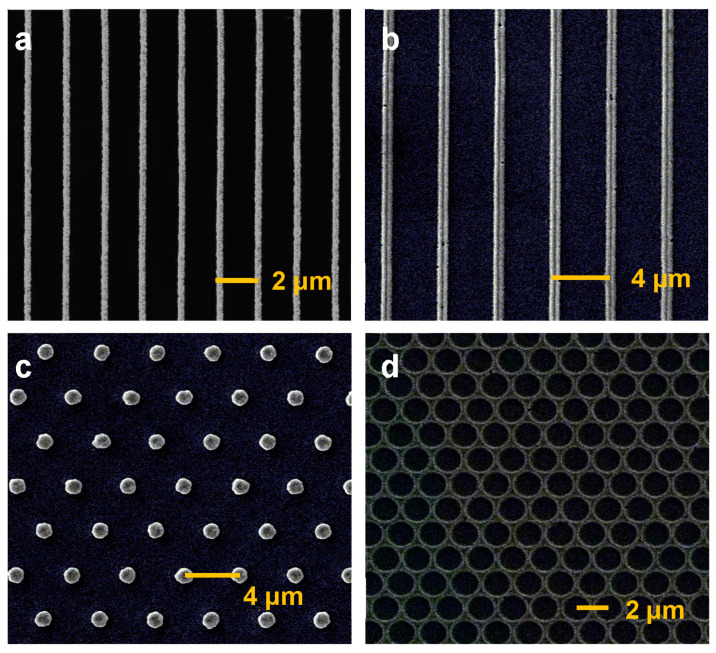
SEM images of homogeneous 1D and 2D gold structures fabricated using our strategy, (**a**) 1D gold grating with the period of 2 μm, (**b**) 1D gold grating with the period of 4 μm, (**c**) 2D gold micron lattice, (**d**) 2D gold honeycomb array.

Figure 4 presents some other gold structures fabricated using our strategy, which shows that the proposed solution-processed direct imprinting strategy is generally applicable to fabricating metallic photonic lattices. For example, the one-dimensional gold grating in Figure 4a,b has a period of 2 μm/4 μm with a gold nanowire width of about 400 nm/800 nm, implying a duty cycle of 20%. It is clear in Figure 4a,b that almost no Au nanoparticles are left in the grooves. Instead, the nanowires are continuous everywhere, and their edges are clear. We further performed atomic force microscopy (AFM) on these two structures (shown in Figure S8): our results demonstrate that the height of the nanowires is around 360 nm/780 nm for the structures in (a) and (b), respectively, implying an aspect ratio close to 1 for these two structures. As for the two-dimensional (2D) structures, Figure 4c shows a 2D gold micron lattice, where the diameter of the gold micro disc is around 1.3 μm, and the distance between the micro discs is around 4.00 ± 0.06 μm. Figure 4d shows a homogeneous 2D gold honeycomb array, where the diameter of pores is around 2 μm, and the distance between pores is around 2.00 ± 0.04 μm. The atomic force microscopy of these two structures (shown in Figure S8) demonstrates that the height of the 2D gold micron lattice in (c) is around 712 nm and the height of the 2D gold honeycomb array in (d) is around 340 nm. These structures are fabricated using an Au nanoparticle coolid concentration of 100 mg/mL with a pressure of 0.25 kg/cm^2^ and an annealing temperature of 300 °C.

## 4. Conclusions

In summary, we have demonstrated a simple, low-cost, solution-processable direct imprinting approach for fabricating metallic photonic lattices with excellent homogeneity over several square centimeters. The optical extinction spectrum measurements indicate well-defined plasmon resonances of individual gold nanowires and strong coupling between the Rayleigh abnormal and the plasmon resonance, illustrating their possible application as infrared plasmonic optical polarization filters. Some other successfully fabricated 1D and 2D metallic photonic lattices using our approach are also presented, demonstrating that this strategy is a significant advance for the high throughput and scalable fabrication of periodic metallic structures. Due to the solution-processed method, we believe our methodology also allows for the fabrication of stretchable and flexible structures in the future, structures to potentially be used in scalable wearable optoelectronic devices.

## Figures and Tables

**Table 1:** Infrared polarization filter parameters at different incident angles.

Incident Angle (°)	Center Wavelength (nm)	Cutoff Wavelength (nm)	Filter Ratio (%)	Bandwidth (FWHM) (nm)
0	1893	1721	88.6	258 ± 2
4	2006	1862	50.7	243 ± 2
8	2113	1994	61.4	286 ± 2
12	2210	2100	61.6	224 ± 2
16	2314	2206	62.3	/

**Table 2:** Comparison of our filters with others reported in the literature.

Angle-Tuning (°)	Center Wavelength (nm)	Filter Ratio (%)	Bandwidth (FWHM) (nm)	Reported Polarization Filters
yes	1893	88.6	258	This work
no	1640	44	79	[29]
no	1540	90	20	[30]
no	900	80	3	[31]
no	6310	57	113	[32]
no	3179	72.6	162	[33]
no	1890	83	111	[34]
no	8480	/	237	[35]
yes	9680–10,970	>90	180–580	[36]
no	2458	80	4	[37]

## Data Availability

The data presented in this study are available on request from the first author.

## Supplementary Materials

The following supporting information can be downloaded at: https://www.mdpi.com/article/10.3390/nano13061022/s1, Figure S1: The SEM image of the cross section of the PDMS mold; Figure S2: SEM images of samples before annealing; Figure S3: a, SEM images of 250 °C annealed gold blazed gratings (period at 1.7 um, pressure is 0.5 kg/cm²) with the Au colloidal concentration of 50 mg/ml, 100 mg/ml, and 150 mg/ml, respectively; b, Optical extinction spectra of these samples at TM mode with the incident angle of 0 degrees; Figure S4: a, SEM images of 250 °C annealed gold blazed gratings (period at 1.7 um, Au colloidal concentration is 150 mg/ml) with the pressure of 0.125 kg/cm², 0.25 kg/cm², 0.5 kg/cm² respectively; b, Optical extinction spectra of these samples at TM mode with the incident angle of 0 degrees; Figure S5: SEM images of 450 ^o^C annealed gold blazed gratings (period at 1.7 um, Au colloidal concentration is 150 mg/ml) with the pressure of 0.125 kg/cm², 0.25 kg/cm², 0.5 kg/cm² respectively; b, Optical extinction spectra of samples at TM mode with the incident angle of 0 degrees; Figure S6: Optical extinction spectra of 400 °C annealed gold blazed grating (period at 1.7 um) at TE and TM mode with the incident angle of 0 degrees; Figure S7: Optical extinction spectra of PDMS mold at TE and TM polarization modes; Figure S8: the AFM images of a–d structures in Figure 4 in the paper.

## References

[1] Zeng B., Gao Y., Bartoli F.J. (2013). Ultrathin Nanostructured Metals for Highly Transmissive Plasmonic Subtractive Color Filters. Sci. Rep..

[2] Neutens P., Lagae L., Borghs G., Dorpe P.V. (2012). Plasmon FIlters and metal-insulator-metal waveguides. Opt. Express.

[3] Wang Y., Zhang X. (2019). Ultrafast Optical Switching Based on Mutually Enhanced Resonance Modes in Gold Nanowire Gratings. Nanoscale.

[4] Gao H., Henzie J., Odom T.W. (2006). Direct Evidence for Surface Plasmon-Mediated Enhanced Light Transmission through Metallic Nanohole Arrays. Nano Lett..

[5] Tvingstedt K., Persson N.-K., Inganas O., Rahachou A., Zozoulenko I.V. (2007). Surface Plasmon Increase Absorption in Polymer Photovoltaic Cells. Appl. Phys. Lett..

[6] Atwater H.A., Polman A. (2010). Plasmonics for Improved Photovoltaic Devices. Nat. Mater..

[7] Gan Q., Bartoli F.J., Kafafi Z.H. (2013). Plasmonic-Enhanced Organic Photovoltaics: Breaking the 10% Efficiency Barrier. Adv. Mater..

[8] Xiang J., Wang Y., Wu Y., Fang H., Shui L., Liu Z., Ding T. (2021). Ordered Hierarchical Ag Nanostructures as Surface-Enhanced Raman Scattering Platforms for (Bio)Chemical Sensing and Pollutant Monitoring. ACS Appl. Nano Mater..

[9] Zhang X., Feng S., Zhang J., Zhai T., Liu H., Pang Z. (2012). Sensors Based on Plasmonic-Photonic Coupling in Metallic Photonic Crystals. Sensors.

[10] Zhang X., Liu H., Tian J., Song Y., Wang L. (2008). Band-Selective Optical Polarizer Based on Gold-Nanowire Plasmonic Diffraction Gratings. Nano Lett..

[11] Shi S., Lu N., Lu Y., Wang Y., Qi D., Xu H., Chi L. (2011). Fabrication of Periodic Metal Nanowires with Microscale Mold by Nanoimprint Lithography. ACS Appl. Mater. Interfaces.

[12] Grande M., Vincenti M.A., Stomeo T., Morea G., Marani R., Marrocco V., Petruzzelli V., D’Orazio A., Cingolani R., De Vittorio M. (2011). Experimental Demonstration of a Novel Bio-sensing Platform via Plasmonic Band Gap Formation in Gold Nano-patch Arrays. Opt. Express.

[13] Grande M., Marani R., Portincasa F., Morea G., Petruzzelli V., D’Orazio A., Marrocco V., De Ceglia D., Vincenti M.A. (2011). Asymmetric Plasmonic Grating for Optical Sensing of Thin Layers of Organic Materials. Sensors Actuators, B Chem..

[14] Ibbotson L.A., Demetriadou A., Croxall S., Hess O., Baumberg J.J. (2015). Optical Nano-Woodpiles: Large-Area Metallic Photonic Crystals and Metamaterials. Sci. Rep..

[15] Garcia J.A., Hrelescu C., Zhang X., Grosso D., Abbarchi M., Bradley A.L. (2021). Quasi-Guided Modes in Titanium Dioxide Arrays Fabricated via Soft Nanoimprint Lithography. ACS Appl. Mater. Interfaces.

[16] Bruchhaus L., Mazarov P., Bischoff L., Gierak J., Wieck A.D., Hövel H. (2017). Comparison of Technologies for Nano Device Prototyping with a Special Focus on Ion Beams: A Review. Appl. Phys. Rev..

[17] Dan V., Dmitruk M., Indutnyi I., Mamykin S., Myn V., Lukaniuk M. (2015). Fabrication of Periodic Plasmonic Structures Using Interference Lithography and Chalcogenide Photoresist. Nanoscale Res. Lett..

[18] Guo L.J. (2007). Nanoimprint Lithography: Methods and Material Requirements. Adv. Mater..

[19] Kothari R., Beaulieu M.R., Hendricks N.R., Li S., Watkins J.J. (2017). Direct Patterning of Robust One-Dimensional, Two-Dimensional, and Three-Dimensional Crystalline Metal Oxide Nanostructures Using Imprint Lithography and Nanoparticle Dispersion Inks. Chem. Mater..

[20] Zhai T., Zhang X., Pang Z., Dou F. (2011). Direct Writing of Polymer Lasers Using Interference Ablation. Adv. Mater..

[21] Chou S.Y., Krauss P.R., Renstrom P.J. (1995). Imprint of Sub-25 Nm Vias and Trenches in Polymers. Appl. Phys. Lett..

[22] Mcdonald J.C., Duffy D.C., Anderson J.R., Chiu D.T. (2000). Review General Fabrication of Microfluidic Systems in Poly (Dimethylsiloxane). Electrophoresis.

[23] Hu Y., Li G., Cai J., Zhang C., Li J., Chu J., Huang W. (2014). Facile Fabrication of Functional PDMS Surfaces with Tunable Wettablity and High Adhesive Force via Femtosecond Laser Textured Templating. AIP Adv..

[24] Nanoparticles S.G., Zhang X., Sun B., Friend R.H. (2006). Metallic Photonic Crystals Based On. Nano Lett..

[25] Zhang X., Liu H., Feng S. (2009). Solution-Processible Fabrication of Large-Area Patterned and Unpatterned Gold Nanostructures. Nanotechnology.

[26] Modaresialam M., Chehadi Z., Bottein T., Abbarchi M., Grosso D. (2021). Nanoimprint Lithography Processing of Inorganic-Based Materials. Chem. Mater..

[27] Halas N.J., Lal S., Chang W., Link S., Nordlander P. (2011). Halas_ChemRev_2011_Plasmons in Strongly Coupled Metallic Nanostructures.Pdf. Chem. Rev..

[28] Liu F., Zhang X. (2015). Fano Coupling between Rayleigh Anomaly and Localized Surface Plasmon Resonance for Sensor Applications. Biosens. Bioelectron..

[29] Shah Y.D., Grant J., Hao D., Kenney M., Pusino V., Cumming D.R.S. (2018). Ultra-Narrow Line Width Polarization-Insensitive Filter Using a Symmetry-Breaking Selective Plasmonic Metasurface. ACS Photonics.

[30] Ng R.C., Garcia J.C., Greer J.R., Fountaine K.T. (2019). Polarization-Independent, Narrow-band, Near-IR Spectral Filters via Guided Mode Resonances in Ultrathin a-Si Nanopillar Arrays. ACS Photonics.

[31] Wang R., Wang R., Gong Q.H., Chen J.J., Chen J.J., Chen J.J. (2020). Extra-Narrowband Metallic Filters with an Ultrathin Single-Layer Metallic Grating. Chin. Phys. B.

[32] Barrow M., Phillips J. (2021). Mid-Wave Infrared Transmittance Filters in Suspended GaAs Subwavelength Gratings. Appl. Phys. Lett..

[33] Shi X., Chen C., Liu S., Li G. (2020). Nonvolatile, Reconfigurable and Narrow-band Mid-Infrared Filter Based on Surface Lattice Resonance in Phase-Change Ge2sb2te5. Nanomaterials.

[34] Chen Y.G., Kao T.S., Ng B., Li X., Luo X.G., Luk’yanchuk B., Maier S.A., Hong M.H. (2013). Hybrid Phase-Change Plasmonic Crystals for Active Tuning of Lattice Resonances. Opt. Express.

[35] Li H.J., Wang L.L., Sun B., Huang Z.R., Zhai X. (2014). Controlling Mid-Infrared Surface Plasmon Polaritons in the Parallel Graphene Pair. Appl. Phys. Express.

[36] Ko Y.H., Lee K.J., Simlan F.A., Gupta N., Magnusson R. (2022). Dual Angular Tunability of 2D Ge/ZnSe Notch Filters: Analysis, Experiments, Physics. Adv. Opt. Mater..

[37] Mao H., Dong X., Liu Y., Silva K.K.M.B.D., Faraone L. (2022). A Suspended Metamaterial Mirror for Hyperspectral Shortwave Infrared Fabry-Perot Filters. J. Microelectromechanical Syst..

