# Supplementary material for: Direct Imprinting of Large-Area Metallic Photonic Lattices for Infrared Polarization Filters with Broadband Tunability
Source: Nanomaterials (Basel). 2023 Mar 12;13(6):1022. doi: 10.3390/nano13061022 (PMC10058146; doi:10.3390/nano13061022)
Supplement: Supplementary file 1 [file nanomaterials-13-01022-s001.zip › nanomaterials-2230800-supplementary.pdf]

nanomaterials

Supporting Information

Direct Imprinting of Large-Area Metallic Photonic Lattices for Infrared Polarization Filters with Broadband Tunability

Fei Dou, Chen Peng, Miaomiao Zou and Xinping Zhang *

College of Physics and Optoelectronics, Faculty of Science, Beijing University of Technology, Beijing 100124, China;
* Correspondence: zhangxinping@bjut.edu.cn

Keywords

Metallic photonic lattices; solution processed direct imprinting; infrared Plasmon resonance; Fano coupling, infrared polarization filters

Highlight

Large-area metallic photonic lattices fabricated by solution processed direct imprinting
Infrared Plasmon resonance in metallic photonic lattices
Fano coupling between Plasmon resonance and Rayleigh anomaly

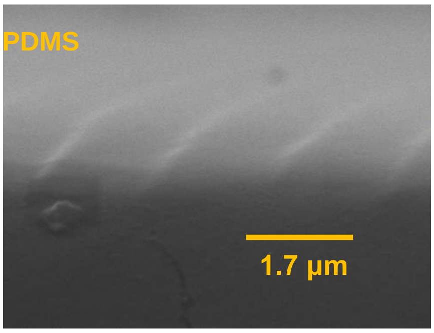

Figure S1. SEM image of the cross section of the PDMS mold.

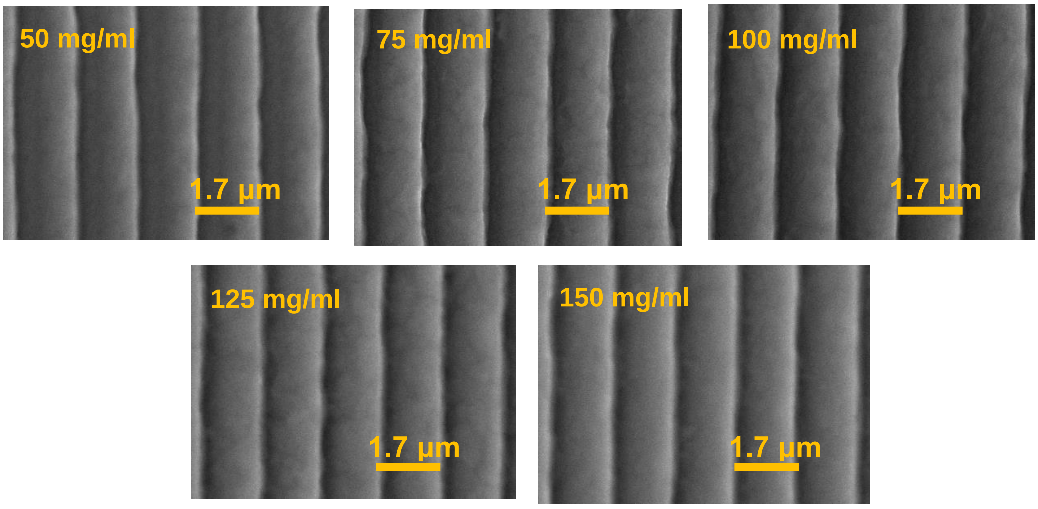

Figure S2. SEM images of samples before annealing (period at 1.7 um, pressure is 0.125 kg/cm^2, Au colloidal concentration varying from 50 mg/ml to 150 mg/ml).

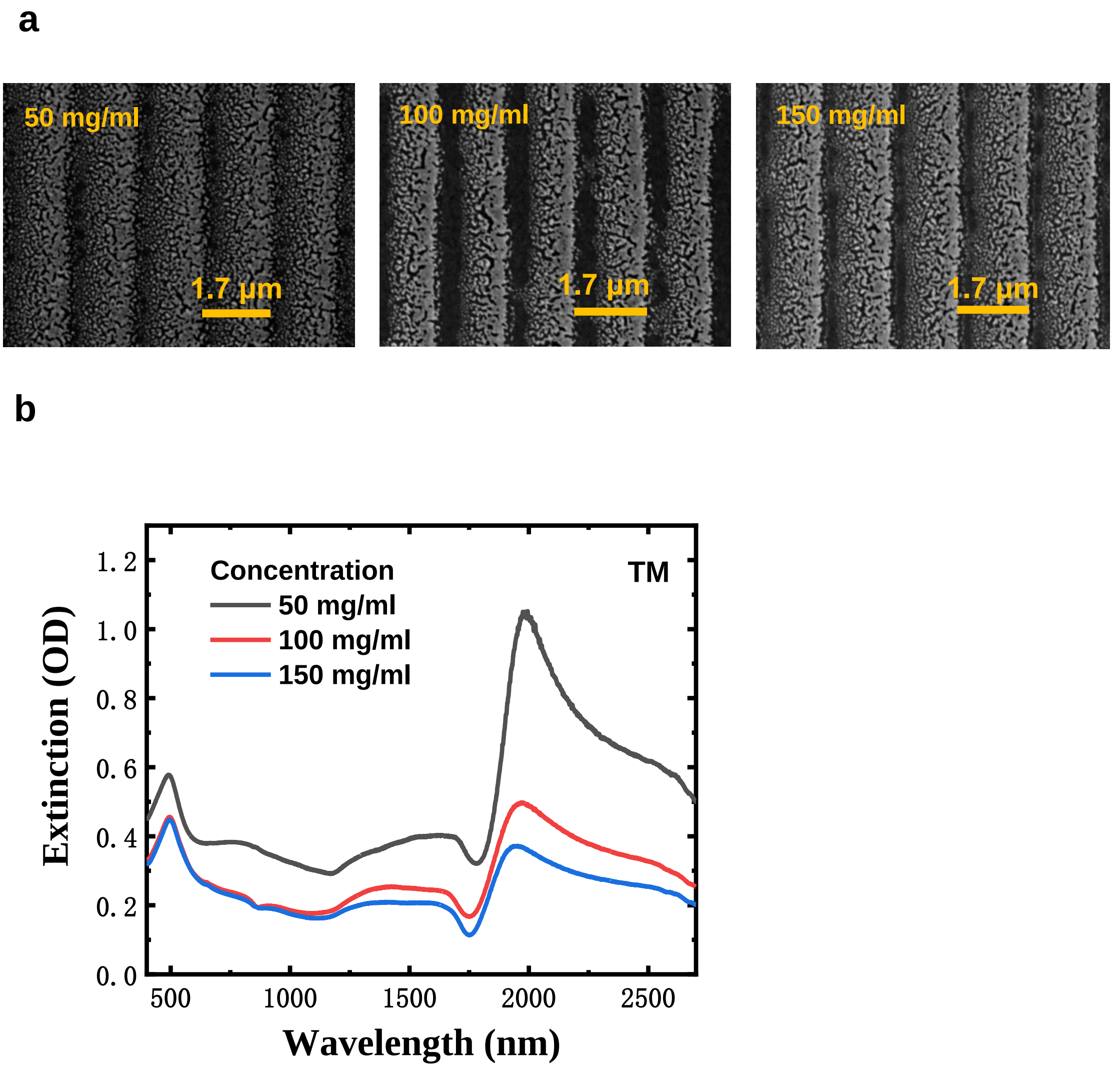

Figure S3.(**a**) SEM images of 250 ^{0}C annealed gold blazed gratings (period at 1.7 um, pressure is 0.5 kg/cm^2) with the Au colloidal concentration of 50 mg/ml, 100 mg/ml, and 150 mg/ml, respectively. (**b**).Optical extinction spectra of these samples at TM mode with the incident angle of 0 degrees. All samples showed Au-aggregate morphology at this annealing temperature. The Plasmon resonance showed up at 2000 nm for all the samples. The 50 mg/ml sample showed the highest extinction intensity.

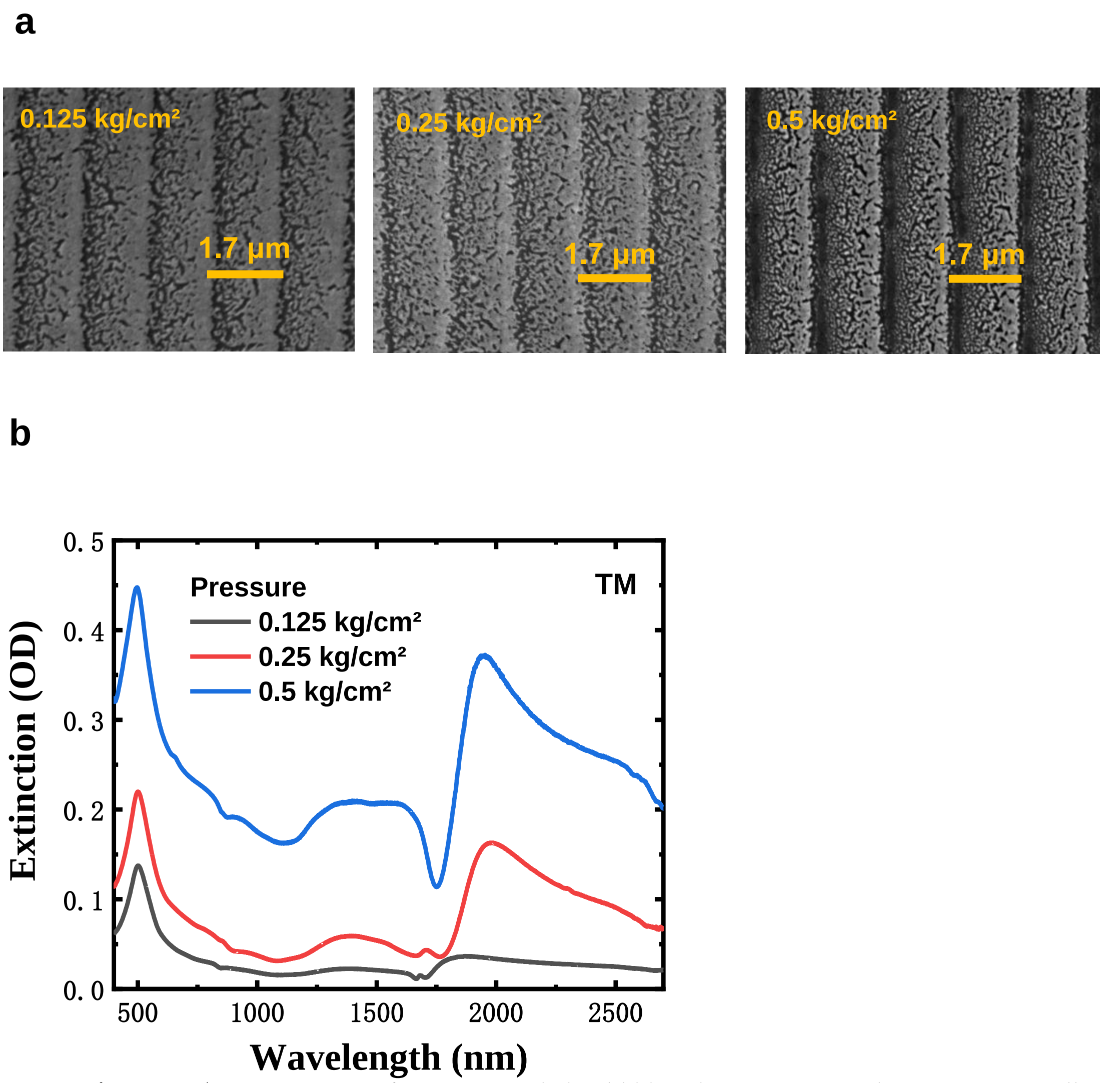

Figure S4. (a) SEM images of 250 ^{0}C annealed gold blazed gratings (period at 1.7 um, Au colloidal concentration is 150 mg/ml) with the pressure of 0.125 kg/cm², 0.25 kg/cm², 0.5 kg/cm² respectively. (**b**).Optical extinction spectra of these samples at TM mode with the incident angle of 0 degrees. All samples showed Au-aggregate morphology at this annealing temperature. The Plasmon resonance showed up at 2000 nm for samples of 0.125 kg/cm² and 0.25 kg/cm². The 0.125 kg/cm² sample showed the highest extinction intensity.

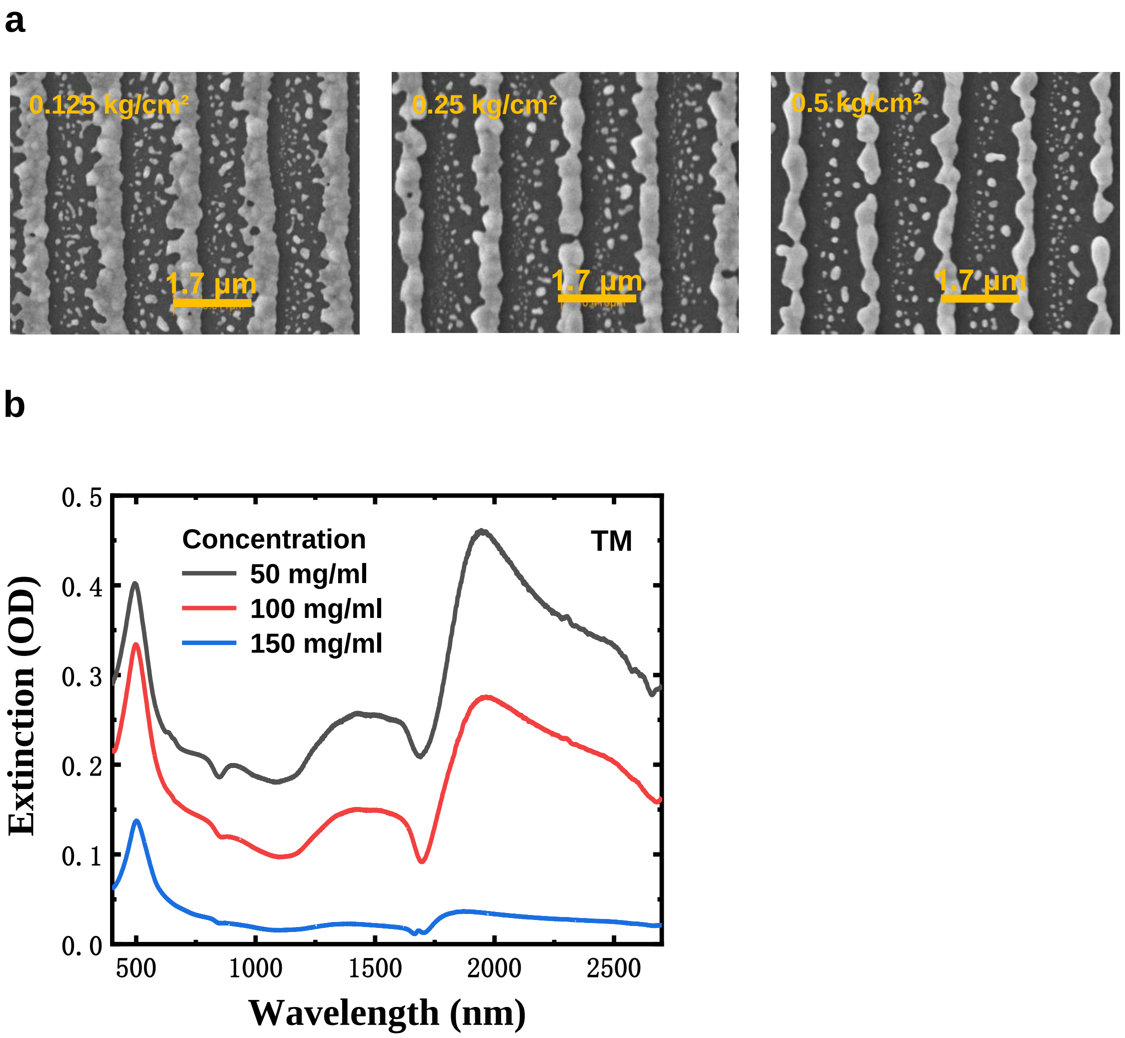

Figure S5. (**a**)SEM images of 450 ^{0}C annealed gold blazed gratings (period at 1.7 um, Au colloidal concentration is 150 mg/ml) with the pressure of 0.125 kg/cm², 0.25 kg/cm², 0.5 kg/cm² respectively. (**b**).Optical extinction spectra of samples at TM mode with the incident angle of 0 degrees. All samples showed Au-bulk morphology at this annealing temperature. The Plasmon resonance showed up at 2000 nm for 50 mg/ml and 100 mg/ml samples. The 50 mg/ml sample showed the highest extinction intensity.

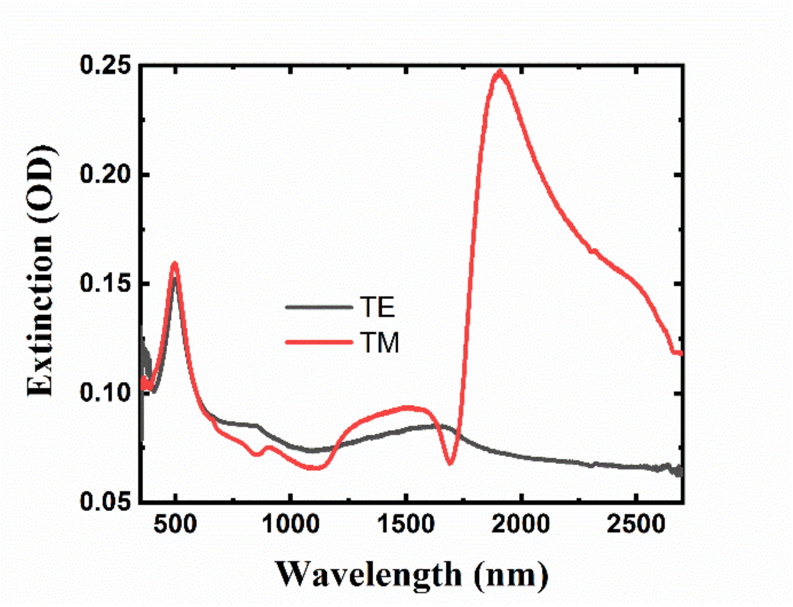

Figure S6. Optical extinction spectra of 400 ^{0}C annealed gold blazed grating (period at 1.7 um) at TE and TM mode with the incident angle of 0 degrees.

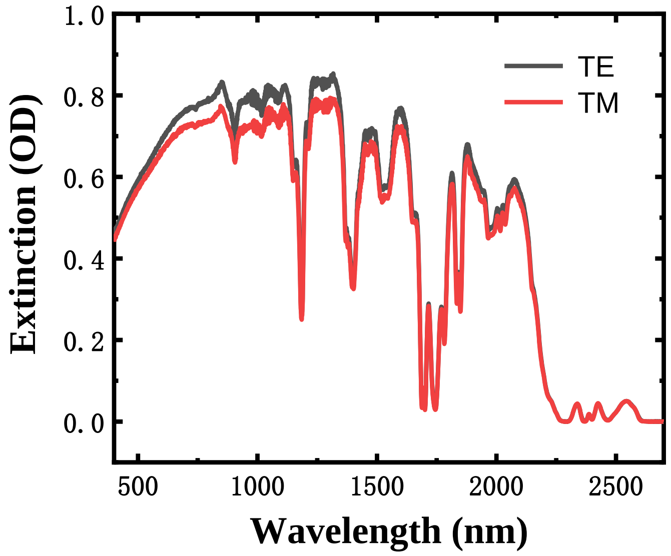

Figure S7. Optical extinction spectra of PDMS mold are similar at TE and TM polarization modes. .

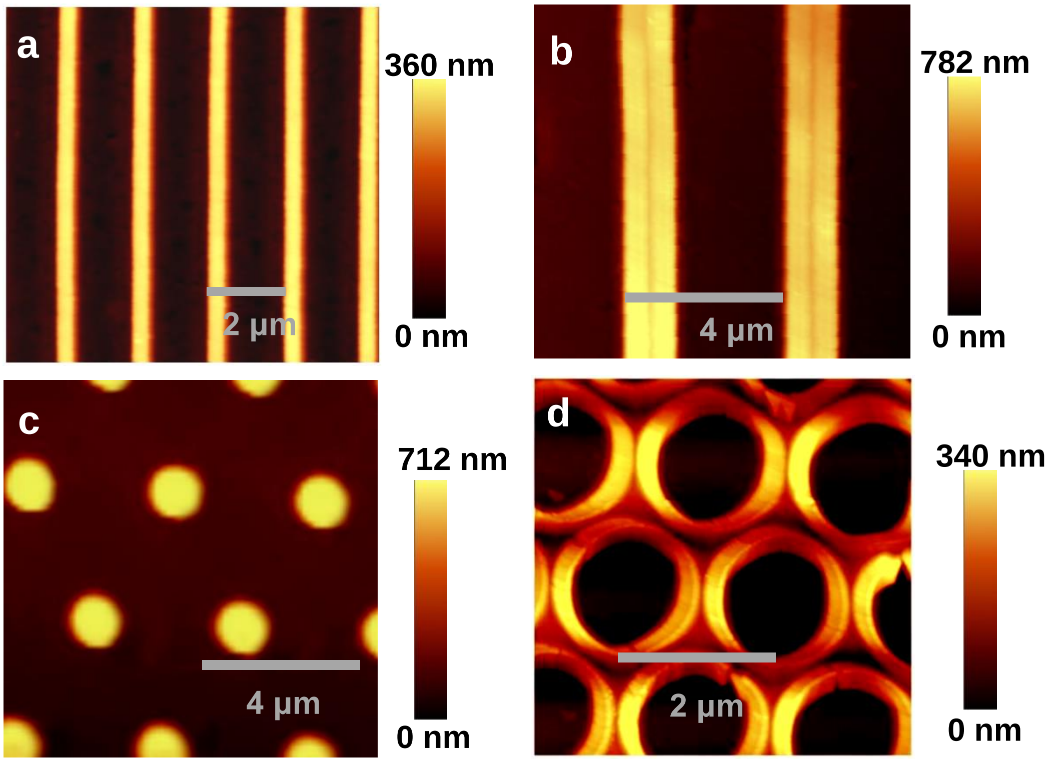

Figure S8. The atomic force microscopic image of a-d structures in **Figure 4** in the paper, results demonstrated the height of nanowires is around 360 nm and 780 nm of 1D structures, respectively, the height of 2D gold micro lattice is around 710 nm, and the height of 2D gold honeycomb array is around 340 nm.
